# Parameterizing Practice in a Longitudinal Measurement Burst Design to Dissociate Retest Effects From Developmental Change: Implications for Aging Neuroscience

**DOI:** 10.3389/fnagi.2022.885621

**Published:** 2022-06-03

**Authors:** Nicholas Tamburri, Cynthia McDowell, Stuart W. S. MacDonald

**Affiliations:** ^1^Brain Aging and Neurocognitive Health Laboratory, Department of Psychology, University of Victoria, Victoria, BC, Canada; ^2^Institute on Aging and Lifelong Health, University of Victoria, Victoria, BC, Canada

**Keywords:** retest effects, practice vs. developmental change, longitudinal measurement burst design, cognitive aging, multilevel modeling (MLM)

## Abstract

**Background**: In longitudinal designs, the extraneous influence of retest effects can confound and obscure estimates of developmental change. The current study provides a novel approach to independently parameterize short-term retest effects and long-term developmental change estimates by leveraging a measurement burst design and three-level multilevel modeling. We further employ these short- and long-term slopes as predictors of cognitive status at long-term follow-up assessments.

**Methods**: Participants included 304 older adults from Project MIND: a longitudinal measurement burst study assessing cognitive performance across both biweekly sessions and annual retests. Participants were classified as either Healthy controls (HC) or Cognitively Impaired, not Demented (CIND) at baseline, the final burst assessment (Year 4), and at an additional four-year follow-up (Year 8). Response time inconsistencies (RTI) were computed at each burst occasion for a simple choice response time (CRT) task and a one-back response time (BRT) task. Three-level multilevel models were employed to simultaneously examine change in RTI for both CRT and BRT across weeks within years, as well as across years, in order to dissociate within-individual retest effects (short-term) from developmental (long-term) change slopes. Individual slopes were then extracted and utilized in a series of multinomial logistic regression equations to contrast short- vs. long-term RTI change as predictors of cognitive status.

**Results**: Separately parameterizing short- and long-term change estimates yielded distinct patterns of variation. CRT RTI remained stable across short-term weekly assessments, while significantly increasing across years. In contrast, BRT RTI decreased significantly across short-term assessments but showed no change across long-term assessments. After dissociating change estimates, short-term BRT as well as long-term CRT and BRT estimates predicted cognitive status at long-term follow-ups; increases in RTI, suggesting either an inability to benefit from retest or process-based developmental decline, were associated with an increased likelihood of being classified as CIND.

**Conclusions**: We showcase an innovative approach to dissociate retest effects from developmental change across and within individuals. Accurately parameterizing these distinct change estimates can both reduce systematic bias in longitudinal trend estimates as well as provide a clinically useful tool by utilizing retest effects to predict cognitive health and impairment.

## Parameterizing Practice in A Longitudinal Measurement Burst Design to Dissociate Retest Effects from Developmental Change: Implications for Aging Neuroscience

The analysis of change has posed numerous seemingly intractable problems for both clinicians and researchers studying human development, prompting contentions as to whether change could, or even should, be measured (e.g., Cronbach and Furby, 1970; Willett, 1988). Such debates motivated a fundamental conceptual shift in which developmental change became viewed as a continuous process that fluctuates over time, as opposed to mere increments between pre-post testing occasions (Willett, 1988). This reconceptualization, paired with Baltes and Nesselroade’s (1979) assertion that one of the primary objectives of developmental research was to directly identify intraindividual change (i.e., exploring within-person processes), facilitated the development of increasingly sophisticated methodologies aimed at providing richer and more accurate parameterizations of between- and within-person change processes. The current study aims to further extrapolate upon these methodologies by employing innovative solutions to some of the more persistent problems inherent in modeling development.

Within aging neuroscience, where developmental outcomes are of central interest, longitudinal designs afford the opportunity to directly observe both age- and process-related change. Such designs allow researchers to avoid the biases inherent in cross-sectional inferences of change (see Baltes and Nesselroade, 1979; Hofer and Sliwinski, 2006; Schaie, 2008)—which employ between-subjects comparisons within age-heterogeneous samples to draw conclusions about the nature of age-graded development—and more appropriately approximate the conceptualization of change as a continuous and oscillatory process (Willett, 1988; Singer and Willett, 2003). However, while advances in conceptual and technical approaches have undoubtedly improved the ability to index change, many problems remain that continue to obfuscate the understanding and measurement of development.

Implicit in the reconceptualization of change as a continuous and intraindividual process is the understanding that change is modulated by a confluence of multiple influences occurring across both short and long temporal intervals. There is a pressing need to dissociate these processes, and their potentially confounding impact on true underlying development, to fully understand moderators of short- and long-term change. Of particular interest, retest effects—changes in performance attributable to previous exposure to the testing materials, environment, and procedures—perturb estimates of aging and development by systematically biasing inter- and intraindividual change trajectories in longitudinal designs (Hoffman et al., 2011). Retest effects, encompassing the more specific delineation of practice effects (i.e., improvements attributable to the repetition of the same or similar materials), are an oft-cited criticism of longitudinal designs and represent an enduring problem in the field of aging neuroscience (e.g., Schaie, 1965; Baltes, 1968). Retest effects have long been known to confound estimates of change across both short- (e.g., between first and second retest occasions) and long-term intervals (e.g., across many years of retest occasions; Thorndike et al., 1928; Ferrer et al., 2004; Wilson et al., 2006; Rabbitt et al., 2009). Given that longitudinal designs offer the only direct way of indexing intraindividual development, overcoming this susceptibility to retest effects is of critical importance to developmental researchers.

Appropriate quantification and parameterization of retest effects are crucial for understanding their unique value as an individual differences predictors. The magnitude of retest effects has shown to be differentiable depending on both test (e.g., complexity, modality) and test-taker characteristics (e.g., IQ, age, personality, mood, motivations; Bartels et al., 2010). The parameterization of retest effects may therefore serve as a useful cognitive variable, indicative of both an individual’s current capacity and predicted cognitive trajectory. While the findings in this domain are equivocal, some evidence suggests that an individual’s ability to benefit from practice is informative of their prospective cognitive health and disease risk—with smaller than expected practice effects in older adults potentially presaging cognitive decline, poorer response to intervention, and greater risk of Alzheimer’s-related pathology (Duff et al., 2017; De Simone et al., 2021). For persons with mild cognitive impairment (MCI), inclusive of amnestic MCI (a-MCI), there is considerably more controversy as to whether these individuals can benefit from retest effects and, if so, across which cognitive domains (see Duff et al., 2017 for review). These contentions are further complicated as there is currently no widely accepted approach for reliably and accurately modeling variance due to retest. However, a recent investigation by De Simone et al. (2021) found that lacking the expected benefits from practice on episodic memory tests was an accurate prognostic indicator of late conversion to Alzheimer’s disease in a-MCI patients. Distinguishing among individuals who will remain stable a-MCI vs. progress to dementia is both a pressing objective and imposing challenge, given the known lability and heterogeneity of this relatively broad cognitive classification (Ganguli et al., 2004; Malek-Ahmadi, 2016). Among other benefits, innovations in parameterizing and dissociating retest from development could facilitate a deeper understanding of the utility of retest effects as sensitive predictors for distinguishing between- and within-person differences in cognitive function.

Formal attempts to control for retest effects have centered upon three basic approaches: (1) materials, (2) research design, and (3) quantitative modeling. A common method of material manipulation used by researchers—the use of alternate forms in cognitive testing—attempts to account for the most basic of practice effects (i.e., repeated exposure to the same testing material). However, this strategy has shown variable effectiveness depending on the construct being tested (Watson et al., 1994; Uchiyama et al., 1995; Benedict and Zgaljardic, 1998) and fails to address issues attributable to the more general impact of retest effects (e.g., previous exposure to the testing environment, procedure, etc.). Therefore, various longitudinal design considerations have been implemented to address this more encompassing definition of retest effects and control for their impact on developmental change.

Traditional longitudinal designs typically consist of widely-spaced measurement occasions (e.g., spanning years) in an effort to capture the timescale by which normative age-graded changes take place. However, in such instances, aging and retest effects are entirely conflated (e.g., 1-year increments in chronological age for a design specifying one-year retest intervals spanning five occasions), posing a particular challenge for modeling distinct and unbiased estimates of either process. Consequently, the failure to account for retest effects often leads to inaccurate characterizations of the rate and pattern of developmental change (e.g., change is underestimated), can cause violations of modeling assumptions (e.g., age convergence), and may undermine subsequent attempts of understanding change through regression or correlation analyses (Sliwinski et al., 2010a). More intricate longitudinal designs, such as waitlist control designs, attempt to address retest effects at the group level by employing a hold-out sample. Thorvaldsson et al. (2006), for example, utilized a waitlist control design to evaluate retest effects within several standardized cognitive performance domains. Initially, the researchers randomly selected one-third of their total sample to be assessed on their cognitive performance between the ages of 70–81. The remaining two-thirds of participants were prescribed as the hold out sample, to be assessed at a later date. From ages 85 to 99 years the cognitive performance of both the participants who were previously assessed (i.e., “original” participants), and a random selection of the remaining two-thirds of participants (i.e., “waitlist” participants), were then assessed concurrently. The comparison of cognitive performance between the original participants and waitlist participants facilitates an estimation of group-level retest effects. However, while this approach reasonably quantifies the average retest effects in a population, it precludes the investigation of intraindividual change and forces researchers to adopt questions of change that accommodate a between-person design (Thorvaldsson et al., 2006; Hoffman et al., 2011). Ultimately, when intraindividual change is of interest, controlling for retest *via* design decisions is exceptionally challenging. Indeed, the nature of repeated-measures data presumes the influence of retest effects as unavoidable (Salthouse’s, 2013) and thus cannot be overcome by study design changes alone. Therefore, in addition to careful design considerations, adept statistical modeling approaches are also needed to more effectively address the impact of retest effects.

Advanced quantitative modeling techniques attempt to parse the effects of retest and aging into separately estimated model parameters. These quantitative approaches frequently consist of hierarchical or more sophisticated computational models (e.g., multilevel modeling, latent growth curve modeling, etc.) that estimate both maturational influences (e.g., aging) along with retest effects as separate parameters within a single analytic model of intraindividual change (e.g., Ferrer et al., 2004; Salthouse et al., 2004; Rabbitt et al., 2008). Although potentially informative, these modeling techniques remain subject to common, underappreciated pitfalls and assumptions that must be explicitly addressed. For instance, satisfying assumptions of age-convergence—that cross-sectional age differences and longitudinal age changes converge onto a common trajectory—is necessary in order to obtain meaningful parameter estimates of aging and retest. Hoffman et al. (2011) assert that failing to test and meet age-convergence assumptions can lead to significant bias and increased Type 1 error rates in the estimation of retest effects. This is particularly the case for traditional longitudinal designs that often leverage equal interval designs where age and retest occasion are perfectly correlated. Disconcertingly, most studies that attempt to directly model retest effects often fail to explicitly test for age-convergence assumptions (Sliwinski et al., 2010b). Furthermore, while retest models attempt to estimate a “test naïve” aging trajectory that is dissociated from retest effects, these models are, in actuality, estimating aging trajectories by holding retest effects constant across time. This implicit assumption, that retest effects are invariant in magnitude across time, is potentially spurious when considering that retest effects are (1) often most pronounced between the first and second measurement occasion (Collie et al., 2003; Bartels et al., 2010; Scharfen et al., 2018), (2) potentially affected by ceiling effects (Calamia et al., 2012), (3) influenced by individual differences in the amount and rate of time-dependent forgetting (MacDonald et al., 2006), and (4) showcase interindividual differences in magnitude dependent on test- and test-taker variables (Bartels et al., 2010). Thus, while the combination of both analytical and methodological advances has clearly informed the extant literature, there are notable gaps remaining vis-a-vis optimal approaches for effectively distinguishing retest effects from change.

Researchers are evidently presented with numerous permutations of both design and analytic strategies that provide differential advantages and disadvantages when investigating longitudinal change in cognition; however, when dissociating and parameterizing retest effects is of critical interest, a recent recommendation suggests combining the advantages of the seldom-used measurement burst design alongside the well-known utility of multilevel modeling (Sliwinski, 2008; Hoffman et al., 2011; Jones, 2015). Measurement burst designs can explicitly measure retest effects by examining variability across both short-term intervals—such as narrowly spaced retests (e.g., daily, weekly) in which meaningful age-based change is unlikely to occur—as well as long-term periods (e.g., yearly) over which durable age-based developmental changes commonly unfold. This design avoids common pitfalls of more traditional longitudinal designs, including concerns of age-convergence and equal-interval measurement occasions, and provides the opportunity for more nuanced statistical analysis. Specifically, multilevel modeling can be used to partial these distinct levels of variability into separate slope parameters, separately estimating and dissociating the impact of short-term retest-related change from more durable developmental change.

Unfortunately, many current investigations of retest effects employ two-level multilevel models for a research objective that is optimally addressed using three-level nested data. Specifically, for measurement burst designs and multilevel modeling to be utilized effectively for modeling retest, the innovative application of three-level multilevel models is required to systematically dissociate variance within-persons across short-term retest occasions (level 1) and long-term developmental intervals (level 2), as well as between-persons (level 3). Investigating two-level models by inappropriately aggregating three-level data not only yields an inaccurate dissociation of retest and developmental change but also generates criticism regarding the leveraging of short-term retest intervals as proxies for retest effects altogether. Salthouse’s (2013), for example, has suggested that employing short-term slopes as indices of retest effects is contingent upon having identified positive, moderately strong associations between short- and long-term change estimates—an intuitive assumption given the expectation that shorter-term retest gains should be positively linked to longer-term developmental increases as well. In contrast to this expectation, Salthouse’s (2013) reported a modest negative association between retest and long-term change in cognition. Notably, however, these findings were based upon a two-level analysis of change (i.e., a latent change analysis) from a data set characterized by at least three nested levels—sessions (level 1), within occasions (level 2), within persons (level 3). Failing to properly account for the nestedness inherent within a dataset can result in parameter estimates that are confounded with extraneous sources of information and violate modeling assumptions (e.g., data dependency) which can result in inaccurate probability estimates and confounded estimates of short- and long-term change. This is especially problematic when the research questions and/or conclusions are predicated upon having accurately quantified variance at select levels. Thus, when considering the viability of using short-term change slopes as indicators of retest effects, utilizing a measurement burst design and a three-level modeling framework will provide a more accurate dissociation and quantification of retest and developmental variance.

Using data from Project Mental Inconsistency in Normals and Dementia (MIND), an innovative longitudinal measurement burst design study, the current study employed advanced quantitative models to dissociate short-term retest effects and long-term developmental change and investigated the relative predictivity of retest and change for differentiating cognitive status subgroups at long-term follow-up assessments. Given that retest and developmental change represent non-independent time structures, we utilized three-level multilevel modeling to separately estimate within-individual change in cognitive function across short-term weekly retests (level 1) and long-term yearly bursts (level 2), as well as between-individual differences (level 3) in cognitive performance. The use of a three-level hierarchical modeling structure, paired with the previously suggested measurement burst design, represents a critical extension of the existing literature that simultaneously parameterizes within-person change across both short-term biweekly assessments (i.e., retest) as well as across longer-term annual assessments (i.e., developmental age-based change). Specific research objectives included: (1) disaggregating short- (weekly) from long-term (annual) change slopes to estimate and empirically evaluate the patterns and association among these estimates of retest and development; and (2) leveraging these dissociable estimates of change, obtained during the course of the 4-year measurement burst study, as independent individual-differences predictors of cognitive status indexed at Year 4 (the conclusion of the burst design) and Year 8 (the conclusion of the Project MIND study). The first objective was accomplished by investigating change in response time inconsistencies (RTI) for two select cognitive measures—a simple choice response time (CRT) task and a more complex 1-back choice response time (BRT) task—using three-level multilevel models. By specifying random effects in these multilevel models, it was possible to derive person-specific change slopes that were extracted to address our second research question which used multinomial logistic regression models to contrast short- and long-term RTI change as predictors of cognitive status at Year 4 and 8 of the study.

Increasing evidence suggests that RTI represents a dissociable dimension of performance relative to mean Response Time (RT) (MacDonald and Stawski, 2015, 2020) that may better capture underlying changes in physiological and cognitive processes (Dixon et al., 2007; de Ribaupierre and Lecerf, 2018). Previous research also suggests that within-person variability is differentially sensitive to cognitive status groups, such that RTI was most pronounced in subjects with more severe cognitive impairment (Strauss et al., 2007; MacDonald and Stawski, 2020). The utilization of RTI is particularly beneficial for the current investigation that leverages lability in cognitive performance—which is particularly sensitive to retest effects and generally resistant to floor and ceiling effects—as a proxy for cognitive health status.

## Materials and Methods

### Participants

Participants were 304 community-dwelling Caucasian older adults aged 64–92 years (*M* = 74.02; SD = 5.95) who were concerned about their cognitive functioning but had not been diagnosed with a neurological disorder. This study was approved by the University of Victoria Human Research Ethics Board and was conducted in accordance with institutional guidelines. Participants (208 female and 96 male) resided in Victoria, Canada and were recruited through local media advertisements (radio and newspaper). Participants were generally well-educated (*M* = 15.15; SD = 3.14; range = 7–24 years of education), performed well on the Mini-Mental State Examination (MMSE; Folstein et al., 1975) (*M* = 28.74; SD = 1.23; range = 24–30), and were in relatively good health (total number of chronic health conditions: *M* = 2.92; SD = 1.91; range = 0–10). Exclusionary criteria at intake included physician-diagnosed dementia or an MMSE score of less than 24, drug or alcohol abuse, psychotropic drug use, current psychiatric diagnosis, a history of significant head injury (e.g., loss of consciousness greater than 5 min), other neurological or major medical illnesses (e.g., Parkinson’s disease, cancer, heart disease), severe sensory impairment (e.g., difficulty reading newspaper-size print, difficulty hearing a normal conversation), and lack of fluency in English.

### Procedure

**Figure 1:**
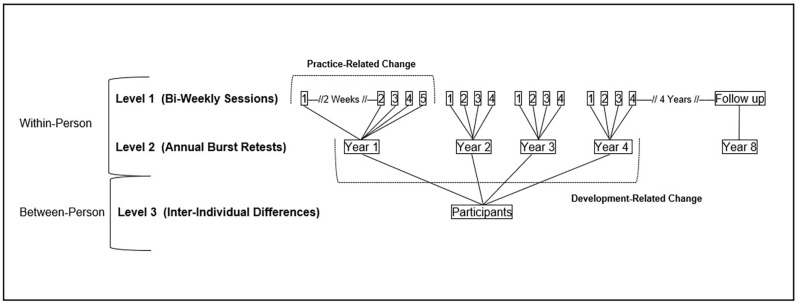
Parameterization of retest and developmental change using three-level multilevel modeling and a measurement burst design.

Participants were initially screened for inclusion and exclusion criteria *via* a telephone interview. Baseline testing occurred across seven sessions (one group and six individuals) scheduled over approximately 3 months. The group testing session was held at the university in our laboratories and the individual testing sessions were conducted in the participant’s home. The first two sessions (one group and one individual) were used to obtain demographic and health information and to administer cognitive measures. Participants then completed a burst evaluation, consisting of five individual biweekly testing sessions that varied across days of the week and times of the day. Within these sessions, participants completed various assessments including cognitive performance measures such as RT tasks that were designed to assess short-term fluctuations in response speed. The entire testing battery was repeated annually four times. During each annual wave, the cognitive measures (inclusive of the burst RT tasks) were identical, and the order of presentation did not vary. However, for each subsequent year after baseline, four (rather than five) biweekly testing sessions were completed, yielding up to 17 total assessments for each individual (see Figure 1). Follow-up demographic and cognitive assessments were then conducted four years following cessation of the burst portion of the study (i.e., at Year 8) to evaluate long-term change in participants’ cognitive status. Eighty percent of participants (*N* = 242) completed all four bursts and attrition rates were 11.0%, 3.5%, and 4.5% of the sample between years 1–2, 2–3, and 3–4, respectively. The attrition rate between Year 4 and Year 8 was 26%, with 61% of the original sample (*N* = 185) completing Year 8.

### Cognitive Status

Cognitive status was ascertained for each year of study according to participant’s performance on five cognitive tasks. The cognitive performance tasks consisted of indicators for perceptual speed (WAIS-R Digit Symbol Substitution; Wechsler, 1981), verbal fluency (Controlled Associations; Ekstrom et al., 1976), vocabulary (Extended Range Vocabulary; Ekstrom et al., 1976), episodic memory (Immediate free word recall; Hultsch et al., 1990), and inductive reasoning (Letter Series; Thurstone, 1962). Participants were classified as cognitively intact healthy controls (HC) or cognitively impaired, not demented (CIND) based upon deficits (1.5 SDs relative to age and education norms) spanning the five distinct cognitive domains. The age and education norms were obtained from 445 adults aged 65–94 years from the Victoria Longitudinal Study (Dixon and de Frias, 2004); this normative comparison sample for deriving cognitive status classifications was partitioned into four age and education groups (age = 65–74 years and 75+ years; education = 0–12 years and 13+ years) with means and standard deviations computed for each of the five cognitive reference measures. Participants classified as CIND were further subdivided as CIND-S based on deficits for a single cognitive measure or as CIND-M based on deficits across two or more of the cognitive reference tasks. A more thorough methodological account of Project MIND, inclusive of the testing and cognitive status classification procedures, can be found in Bielak et al. (2010).

### Response Time

RT tasks were presented on a Panasonic CF-48 laptop computer (Intel Pentium III 800-MHz processor, MS-DOS operating system Version 4.10.2222) with a 14” color screen. The computer processor controlled the stimulus presentation and timing for each RT task. Participants responded to stimuli by pressing keys on a custom-designed response console consisting of an aluminum enclosure encompassing four response keys in a linear array. This response box was interfaced with the laptop through a PCMCIA Game Port, directly accessible by the CPU, in order to ensure millisecond timing latency (±1 ms). The RT tasks were programmed using C++ and were run on MS-DOS.

### Choice Response Time (CRT)

Participants were presented with four plus signs displayed in a horizontal row along with a response input device containing four spatially-mapped keys. On each trial, following a 1,000 ms delay, a box replaced one of the plus signs. For each trial, participants were asked to respond to the location of the box as quickly as possible. Ten practice trials were followed by 60 test trials. The response latencies of the 60 test trials were used for analysis (Bielak et al., 2010).

### One-Back Choice Response Time (BRT)

The BRT task used the same display, response box, and stimulus presentation design as the CRT task. However, for each trial, participants were asked to respond to the location of the box on the “previous” trial. A total of 10 practice trials followed by 61 test trials were administered. As participants did not respond on Trial 1, it was omitted and only the response latencies of the remaining 60 test trials were used for analysis.

### Data Preparation

#### Outliers and Missing Values

All RT data were examined for outliers by examining the distributions of raw latency scores at the individual level. Exceptionally slow or fast responses were removed and considered likely to represent sources of measurement error (e.g., accidental key press). Valid lower bound response times have been provided by previous research (150 ms; Hultsch et al., 2002), and valid upper bounds were identified by calculating intraindividual means and standard deviations for each task and measurement occasion; for each individual, any trials that exceeded their personal mean by three or more standard deviations were removed. For each of the CRT and BRT tasks at Year 1, a total of 91,200 trials were possible across individual assessments (60 trials per administration of each RT task), sessions (five biweekly retests), and persons (*n* = 304; 60 × 5 × 304 = 91,200). For the CRT task, 0.13% of trials were excluded due to missing values, 1.43% due to incorrect responses, and 1.78% due to trimming outliers, leaving 96.65% usable trials. For the BRT task, 0.20% of trials were excluded due to missing values, 10.46% due to incorrect responses, and 2.42% due to trimming outliers, leaving 86.93% usable trials. This data preparation procedure for eliminating outliers represents a conservative approach to examining intraindividual variability in RT performance by reducing within-subject variation.

#### Computation of Response Time Inconsistency (RTI)

RTI was indexed using residualized intraindividual standard deviation (ISD) estimates. The residualized ISD estimates were computed across RT trials for each session and burst, residualizing select confounds from the raw data by fitting a multilevel model in order to dissociate within- and between-subject sources of variation (MacDonald and Stawski, 2020). Removing systematic confounds yields RTI estimates that are not conflated with mean age differences in response speed, developmental change, or practice effects at the trial-to-trial level (Stawski et al., 2019; MacDonald and Stawski, 2020). For each session and burst, the computed residualized ISD scores were then linearly transformed into standardized T scores (*M* = 50, SD = 10). See Hultsch et al. (2008) for a full description of this procedure.

### Statistical Procedure

The nested three-level data structure for the present study is characterized by weekly assessments (level 1) nested within annual bursts (level 2) nested within persons (level 3). Using the “nlme” package in R (Pinheiro et al., 2022), we addressed the first research objective by fitting three-level multilevel models to predict change in RTI for both CRT and BRT across sessions (biweekly assessments), bursts (annual retests), and persons. Multilevel modeling decomposes total variability into within- vs. between-person sources. Moreover, this multilevel framework, coupled with the current measurement burst design, facilitates parsing of intraindividual variability from intraindividual change (Nesselroade, 2002), thereby separately yet simultaneously indexing retest effects and developmental change, respectively.

Variance decomposition in CRT and BRT RTI across weeks, years, and persons was based upon preliminary fully unconditioned models. Two independent, conditioned longitudinal models were then fit to examine change in CRT and BRT RTI separately. Equation 1 demonstrates the modeling of average linear change in CRT RTI as a function of weekly and yearly assessments (fixed slope effects) and the variability of change across individuals (random slope effects). Response time inconsistencies on the CRT task (CRT RTI_ijk_), for a given week (i), year (j), and person (k), were modeled as a function of that individual’s performance at baseline testing, plus their average individual rate of change per each additional week and year examined (the slopes), plus an error term (ε). A number of random effects were also modeled, with the level-1 residuals [Var(ε_ijk_)] reflecting within-person week-to-week variability, and the level-2 residuals [Var(μ_0jk_)] indexing within-person variability across the annual retest bursts. Variance in the level-3 residuals [Var(υ_00k_)] index between-person stable variability averaged across all biweekly retests and annual burst assessments. Select fixed effects of interest include population estimates for the average CRT RTI score (γ_000_), the average biweekly retest (i.e., practice) effect (γ_100_) as well as the average yearly retest (i.e., long-term developmental change) effect (γ_010_).

**Equation 1**


$$Level\;1\;CRT\;RTI_{ijk}=\beta_{0jk}+\beta 1_{jk}Week_{ijk}+\varepsilon_{ijk}$$

$$\begin{array}{l} Level\;2\;\beta_{0jk}=\delta_{00k}+\delta_{01k}Year_{jk}+\mu_{0jk}\\ \;\beta_{1jk}=\delta_{10k}+\mu_{1jk} \end{array}$$

$$\begin{array}{l} Level\;3\;\delta_{00k}=\gamma_{000}+\gamma_{001}Age_{k}+\gamma_{002}Sex_{k}+\upsilon_{00k}\\ \;\delta_{01k}=\gamma_{010}+\gamma_{011}Age_{k}+\gamma_{012}Sex_{k}+\upsilon_{01k}\\ \;\delta_{10k}=\gamma_{100}+\upsilon_{01k} \end{array}$$

Weekly (Level 1) and yearly (Level 2) linear effects were centered at baseline (e.g., the first week for Year 1). Person (Level 3) covariates included age at baseline (γ_001_; centered at age 74) and sex (γ_002_; centered as 0 = males/1 = females) Parameter estimates were derived using full information maximum likelihood (FIML) estimation, using all available data under the assumption of missing at random (MAR; Grand et al., 2016).

For the second research question, we employed polytomous (multinomial) logistic regression to examine changes in RTI (both short-and long-term) as predictors of cognitive status in Year 4 and Year 8. Healthy controls served as the referent group for each model. Due to the small values of bi-weekly change (i.e., retest) estimates for both CRT and BRT (a consequence of millisecond temporal scaling), we rescaled these values as seconds to facilitate the interpretability of model point estimates and odds ratios.

## Results

### Patterns of Retest and Development Across Time

Sample characteristics are reported in Table 1. To address the first research objective, and to provide an index of the data dependency inherent in our repeated measures design, unconditional models were first fit to decompose the total variability into within-person (weekly and yearly) and between-person sources. Of the total variability in CRT RTI across the sample, 68% reflected variability between-persons, whereas 6% and 26% reflected within-person variability across years and weeks, respectively. Comparable patterns were found for BRT RTI in which 75% of the total variability was between-persons, 7% within-persons across years, and 18% within-persons across weeks.

**Table 1:** Sample characteristics as a function of baseline cognitive status.

	HC	CIND-S	CIND-M
Baseline	*N* = 136	*N* = 88	*N* = 80
Age (years)	73.3 (5.4)	73.8 (6.0)	75.5 (6.6)
Sex (% males)	29	26	43
Education (years)	15.9 (3.1)	15.2 (3.1)	14.3 (3.2)
MMSE score	29.0 (1.0)	28.7 (1.1)	28.3 (1.5)
^a^Medications	5.8 (3.5)	5.4 (3.3)	6.5 (8.9)
^b^Risk Factor (% without)	84	83	73
CIND Classification Year 4	*N* = 138	*N* = 62	*N* = 45
CIND Classification Year 8	*N* = 112	*N* = 40	*N* = 33

*^a^Self-reported number of total prescribed medications. ^b^Presence of risk factor (Significant Hearing Loss, Neurological and/or Cardiac Condition). HC, Healthy Controls; CIND-S, Cognitively Impaired, not Demented based on single task deficit; CIND-M, Cognitively Impaired, not Demented based on >2 task deficits; MMSE, Mini-Mental State Examination (Folstein et al., 1975)*.

Conditioned longitudinal change analyses were then fit using three-level multilevel models to dissociate retest (i.e., short-term) from developmental (i.e., long-term) change estimates. Specifically, these models derived separate estimates of within-person change across both weeks and years, with the former estimate indexing change due to retest and the latter change due to developmental processes (see Figure 1). Between-person differences at baseline and across years were also explored, with random intercept and slope effects estimated to facilitate the derivation of individual slopes for use in subsequent logistic regression equations.

Two separate models, controlling for age and sex, were fit to evaluate CRT RTI and BRT RTI independently as cognitive outcomes. Analyses revealed notable differences between retest and developmental change parameter estimates within each model, as well as between the two models. Specifically, population estimates for the CRT outcome model indicated non-significant short-term change in RTI (*β* = -0.02, *p* > 0.05), with this stability across week-to-week assessments connoting the absence of practice effects. However, RTI significantly increased across years in the study (*β* = 0.15, *p* = 0.005), demonstrating increasing cognitive variability in CRT performance over longer developmental trajectories. In contrast, our BRT model yielded an inverse pattern, perhaps reflecting the inherent differences in cognitive demands between the BRT and CRT measures. Within BRT, a task that requires higher-order cognitive processes (e.g., executive functioning), significant short-term declines in RTI (*β* = -0.06, *p* < 0.0001) exemplified the expected benefits of practice in reducing performance inconsistencies across week-to-week assessments. Yet, non-significant change in long-term RTI slopes (*β* = −0.09, *p* > 0.05) demonstrated stability in patterns of BRT consistency across years. Regardless of RT task, short and long-term change slopes—reflecting the presence of retest vs. developmental change—yielded distinct sources of information. Of note, age significantly predicted between-person differences in both the CRT (*β* = 0.21, *p* < 0.001) and BRT (*β* = 0.28, *p* < 0.001) tasks, such that increasing age resulted in increased inconsistencies for each RT task. Sex did not significantly predict RTI (*p* > 0.05) in either model. The fixed effects from these conditioned models are displayed in Table 2.

**Table 2:** Fixed effects for CRT and BRT three-level multilevel models.

	CRT RTI			BRT RTI		
*Predictors*	β	CI	*p*	β	CI	*P*
Intercept	7.44	6.91–7.96	<0.001	7.23	6.57–7.89	<0.001
Short-Term	−0.02	−0.05–0.00	<0.082	−0.06	−0.09 to −0.04	<0.001
Long-Term	0.15	0.05–0.25	<0.005	−0.09	−0.02–0.02	<0.124
Age	0.21	0.16–0.26	<0.001	0.34	0.28–0.40	<0.001
Sex	−0.24	−0.87–0.38	<0.439	0.25	−0.54–1.04	<0.534

### Retest and Developmental Change as Predictors of Cognitive Status

The aforementioned results summarized our fixed effects which describe the aggregated rates of change in the sample. However, we also estimated random effects in order to derive individual estimates of short- and long-term change for use as predictors of cognitive status. Specifically, to assess whether individual differences in retest effects and developmental change predicted cognitive status at long-term follow ups (Years 4 and 8), person-specific residuals were used to derive individual slope estimates for entry as predictors in several multinomial logistic regression models. These models investigated whether individual differences in short- and long-term rates of change in CRT and BRT RTI were predictive of CIND status upon conclusion of the burst portion of Project MIND (Year 4), as well as at the termination of the study (Year 8). At both Year 4 and Year 8 follow-up assessments, four separate multinomial logistic regression models, controlling for age and sex, were fit for each of our four RTI-related predictors: short-term CRT RTI, short-term BRT RTI, long-term CRT RTI, and long-term BRT RTI. These models were fit independently to avoid potential issues of collinearity between the short- and long-term slope estimates within each cognitive measure. Parameter estimates for these RTI predictors are presented in Table 3.

**Table 3:** Multinomial logistic regression: weekly and annual RTI in relation to the likelihood of cognitive impairment status at Year 4 and Year 8.

	Year 4						Year 8					
	**CIND-S**			**CIND-M**			**CIND-S**			**CIND-M**		
*Variable*	OR	95% CI LB	95% CI UB	OR	95% CI LB	95% CI UB	OR	95% CI LB	95% CI UB	OR	95% CI LB	95% CI UB
Weekly CRT RTI	1.36	0.80	2.34	1.16	0.62	2.17	1.78	0.93	3.40	1.30	0.61	2.77
Yearly CRT RTI	1.81	0.76	4.34	4.33*	1.70	11.05	1.55	0.48	4.98	0.93	0.27	3.22
Weekly BRT RTI	2.26*	1.31	3.88	3.82**	2.14	6.84	1.94	0.99	3.81	2.50*	1.26	4.98
Yearly BRT RTI	2.05*	1.16	3.62	3.10**	1.70	5.68	1.68	0.81	3.51	2.28*	1.04	4.77

Note. Age is baseline age centered at 74 years. Sex is categorically coded with females (1) as the reference category. Healthy controls (HC) serve as the reference category. CIND-S, cognitively impaired-not demented for a single cognitive outcome; CIND-M, cognitively impaired-not-demented for two or more cognitive outcomes; LB, lower bound; UB, upper bound; CRT, choice reaction time; BRT, 1-back choice reaction time; RTI, response time inconsistency. *p < 0.05; **p < 0.001.

Short-term change slopes in CRT, indexing retest effects in the present study, were not significantly predictive of CIND status at either Year 4 or Year 8. However, short-term practice-related gains in BRT RTI were significantly associated with an increased likelihood of being classified as CIND-S [OR = 2.26, 95% CI (1.31, 3.88), *p* = 0.003] and CIND-M [OR = 3.82, 95% CI (2.14, 6.84), *p* < 0.001] at Year 4, as well as CIND-M at Year 8 [OR = 2.50, 95% CI (1.26, 4.98), *p* = 0.009].

In contrast, elevated yearly RTI was associated with increased odds of being classified as CIND relative to HC for both CRT and BRT. Long-term developmental slope estimates for CRT RTI were significantly associated with increased odds of being classified as CIND-M [OR = 4.33, 95% CI (1.68, 11.05), *p* = 0.002] at Year 4, with no significant associations at Year 8. Thus, holding constant age and sex differences, year-to-year unit increases in CRT RTI increased the likelihood of being classified as CIND-M over healthy controls by 333 percent. Additionally, unit increases in yearly BRT RTI were associated with an increased likelihood of being classified as CIND-S [OR = 2.05, 95% CI (1.16, 3.62), *p* = 0.014] and CIND-M [OR = 3.10, 95% CI (1.70, 5.68), *p* < 0.001] at Year 4, as well as CIND-M at Year 8 [OR = 2.23, 95% CI (1.04, 4.77), *p* = 0.039].

To further inform these patterns, four separate multinomial logistic regression models were fit using person-level baseline MMSE scores to contrast the predictivity of long-term cognitive status with our residualized RTI slope parameters. Specifically, we were interested in identifying whether a simple baseline cognitive measure would significantly contribute to model fit or show comparatively accurate long-term predictions of cognitive health status. Across all models, baseline MMSE performance neither significantly contributed to model fit nor predicted cognitive status at long-term follow ups, underscoring the utility of retest effects as more sensitive prognostic indices of cognitive health.

Our models also identified age as a significant predictor of CIND status, with increasing age generally facilitating an increased likelihood of being classified as cognitively impaired. Specifically, at Year 4, age significantly predicted both CIND-S and CIND-M for three of four models (with the exception of yearly CRT RTI which predicted CIND-M only). At Year 8, age was a significant predictor of CIND-M only, regardless of RT task or weekly or yearly RTI. Depending on the model, older age significantly predicted cognitive status such that each additional year beyond age 74 resulted in a 5%–10% increased likelihood of cognitive impairment, relative to controls. Sex (male or female) did not significantly predict cognitive status in any of the eight models.

Finally, to further delineate associations between individual slopes of short- and long-term change, we computed simple bivariate correlations for both CRT and BRT RTI. Correlations between short- and long-term individual BRT RTI slopes were significant and strong at the two-tailed level (*r* = 0.87, *p* < 0.001). For CRT RTI, short- and long-term change slopes shared a more modest but still significant association (*r* = 0.39, *p* < 0.001).

## Discussion

The current investigation showcases an innovative approach for studying practice effects in community-dwelling older adults using both novel design considerations and advanced statistical methodology. By utilizing a measurement burst design—in which data were collected across weeks within years, as well as across years—and employing three-level multilevel models, we were able to (a) dissociate short-term retest effects from long-term developmental change, (b) demonstrate that within-person change across these varying temporal intervals yields distinct patterns of variation, and (c) leverage these retest and change slopes as predictors of cognitive impairment status. The difference in slope estimates between short- and long-term change, and their respective predictive utility, highlights both (a) the advantage of the current approach for dissociating retest effects from developmental change, as well as (b) the promise of employing retest as a proxy for individual differences in cognitive health.

### Retest Can Bias Estimates of Developmental Change

An enduring criticism of longitudinal research concerns the presence of retest effects which may obfuscate the magnitude, shape, and even estimated direction of developmental change. Although retest-related gains are thought to bias development and age-related changes in cognitive performance (Wilson et al., 2006; Hoffman et al., 2011; MacDonald and Stawski, 2020), retest effects are seldom systematically measured or controlled for, due in part to the limitations of existing designs and quantitative methodologies (Sliwinski and Mogle, 2008; Salthouse’s, 2013). Therefore, novel longitudinal approaches that consider the impact of retest effects and the utilization of advanced modeling approaches are needed to adequately distinguish within-person developmental change from retest-related change.

We investigated the extent to which weekly change (i.e., influenced by retest effects) and yearly change (i.e., influenced by aging and development) reflect comparable or distinct sources of information. Consistent with expectations, distinct and divergent patterns were present between the weekly short-term and annual long-term change slopes in both RT tasks. Non-significant, stable change in RTI in the CRT task over short retest intervals was differentiated from significant long-term performance declines. This is consistent with the understanding that simple psychomotor abilities (e.g., sensorimotor speed, processing speed) are less susceptible to the influence of retest and showcase normative declines with aging (Salthouse, 1996; Duff et al., 2017). For RTI in the BRT task, our sample showed the expected benefits of retest with significant short-term performance gains but demonstrated non-significant change over longer retest intervals. These patterns are also congruent with previous research, as the BRT task—which draws upon more executive processes (e.g., updating)—is increasingly susceptible to practice-related gains pursuant to repeated exposure (Grand et al., 2016). The use of such a task helps bolster the idea that placing more demands on cognitive processing resources may provide a more sensitive evaluation of retest effects. Such disparate patterns observed in the fixed effects for both the simpler CRT task, and the more cognitively demanding BRT task, indicate that the within-person change slopes across weekly and annual temporal intervals reflect non-redundant sources of information. Neglecting to parse cognitive performance according to these distinct time structures would bias slope estimates, confounding retest effects with developmental change. These results corroborate previous research demonstrating the important and considerable impact of retest on developmental change slopes (e.g., Wilson et al., 2006; Hoffman et al., 2011; Jones, 2015) and suggest that related but unique associations exist between these constructs. Moreover, overlooking the potential influence of retest effects may mask underlying cognitive symptomatology or early detection of cognitive decline.

The non-significant developmental slope in BRT RTI may, despite our systematic parsing of short-term retest-related variance from long-term parameter estimates, be indicative of the more enduring, generalized impact of retest—which has shown to exert influence across much longer retest (e.g., years) intervals (Rabbitt et al., 2004; Salthouse et al., 2004). However, an alternative explanation is that the observed long-term BRT RTI stability is a consequence of collapsing individual performance information across all cognitive status groups onto one linear trajectory. The heterogeneity in cognitive status produces diverging trajectories of RTI among CIND subgroups (see MacDonald and Stawski, 2020), yet yields a relatively flat sample average slope when combined. Notably, the shape and magnitude of the sample average slope are less consequential to our key research focus, which is concerned with evaluating whether a) there are individual differences in short- and long-term change, and b) these individual differences in slopes are linked to cognitive status. Therefore, the choice to model the data as an average slope, irrespective of cognitive status (i.e., not including a CIND status moderator), was intentional in order to derive person-specific slopes (reflecting individual deviations in change from the population average) which could predict cognitive status at long-term follow-ups.

### Utilizing Retest as a Predictor of Prospective Cognitive Impairment

The focus of our second research objective was to investigate whether the unique intraindividual slopes derived from our models were predictive of cognitive health outcomes (i.e., CIND), for as many as four years following the completion of cognitive testing. We examined within-person change directly by investigating whether an individual’s short-term retest slope predicted long-term cognitive status, and whether their developmental slope reflected a reliable index of process-based change (dissociable from short-term change). A series of multinomial logistic regression models were used to contrast short- vs. long-term CRT and BRT RTI as individual predictors of cognitive status at the final burst assessment wave (Year 4) as well as an additional four years later at the conclusion of the study (Year 8). These follow-up assessments correspond with the natural middle and endpoints of the study and afford a novel opportunity to investigate the differential predictive utility of our discrete cognitive slopes.

Using this approach, we demonstrated that the likelihood of being classified as CIND-M relative to HC at Year 4 was over three times greater for individuals showcasing annual increases in CRT RTI. This result, including the non-significant predictive ability of short-term CRT RTI change, is consistent with the extant literature on psychomotor function and decline. Specifically, the basic sensorimotor demands of the comparatively less cognitively demanding CRT task resulted in less intraindividual variability and diminished retest from which to accurately predict long-term cognitive status. However, interindividual differences in annualized intraindividual change may be reflective of unique intraindividual processes (e.g., normative or pathological aging) or characteristics (e.g., health-related comorbidities) that facilitate more accurate predictions of cognitive impairment status at long-term follow-up (Stawski et al., 2015). Although annualized CRT RTI was not significantly predictive of cognitive status at Year 8, this may be due in part to the relative heterogeneity and lability of CIND classifications or the relative insensitivity of developmental CRT RTI as a proxy for underlying bio-cognitive dysfunction.

In contrast to CRT RTI, increases in intraindividual BRT RTI across both weeks and years were significantly predictive of cognitive status at Years 4 and 8. These patterns reflect the expected influence of both retest and developmental performance on long-term cognitive status. Individuals who failed to benefit from retest and exhibited increases in their short-term BRT RTI were significantly more likely to be classified as CIND-S or CIND-M at Year 4, as well as CIND-M at Year 8. These predictive patterns support the potential clinical utility of retest, where the ability to benefit from practice is postulated to be a function of underlying cognitive health (Galvin et al., 2005; Duff et al., 2011, 2012). Long-term increases in annual intraindividual BRT RTI were also associated with increased odds of being classified as CIND-S and CIND-M at Year 4, and CIND-M at Year 8. Independent of age and sex differences, individuals characterized by increasing BRT RTI across short- and long-term intervals were associated with an increased likelihood of cognitive impairment classification. The identical trends between weekly and annual increases in BRT RTI underscore a key finding of our study: when appropriately parameterized, both intraindividual retest and developmental change slopes can yield distinguishable and meaningful predictions of long-term health outcomes.

### Retest as an Early Indicator of Cognitive Decline

The observed discrepancies between the CRT and BRT tasks are consistent with previous research indicating that retest effects are test-specific (Benedict and Zgaljardic, 1998; Wilson et al., 2006). In comparison to the CRT task, the BRT task involves increased cognitive demands that likely involve attention, working memory, and inhibitory control which are more sensitive to retest effects (Grand et al., 2016). This dependency on executive processes not only underscores why BRT RTI is more sensitive to retests effects but may also help elucidate why both short- and long-term BRT performance showcased greater predictive accuracy for classifying CIND status at Year 8.

More generally, RTI holds considerable promise as a sensitive marker of normal and pathological aging and has received much attention for its promise as a proxy for central nervous system (CNS) health and an early indicator of cognitive impairment or decline (Hultsch et al., 2000; Bielak et al., 2010; MacDonald et al., 2011; MacDonald and Stawski, 2020). RTI has been shown to predict late-life deleterious health outcomes (e.g., fall risk, vascular impairment, dementia; for review, see MacDonald and Stawski, 2015) and may enhance our understanding of the dynamic relationship between individual fluctuations in cognitive performance and underlying CNS integrity (Halliday et al., 2017). RTI has also garnered empirical support as an indicator of lapses of attention (particularly for tasks requiring executive control processes; West et al., 2002), processing efficiency (Eysenck and Calvo, 1992; Brose et al., 2010), and has been shown to fluctuate depending on perceived competence in cognitive control (e.g., individual differences in control beliefs for age-related changes in cognitive performance). For example, in a recent investigation of RTI in both CRT and BRT measures, Cerino et al. (2020) identified that increases in perceived competence were associated with lower RTI on the CRT task, and higher (i.e., maladaptive) RTI performance on the BRT task in older adults. Taken together, BRT RTI may serve as a unique cognitive health indicator, sensitive to disruptions in executive function attributable to both labile (e.g., momentary fluctuations in attention reflecting mental noise, daily variations in sleep or distress) or more chronic mechanisms (e.g., pathological aging, dopaminergic dysregulation, declining CNS signaling fidelity) affecting higher-order cognition. In the context of the present study, increased RTI for the BRT (vs. CRT) task may be a more effective proxy for these underlying bio-cognitive disturbances, which may account for BRT’s increased predictivity at both the level of retest and development across longer time periods.

Accordingly, RTI across both short-and long-term follow-up intervals demonstrated stronger predictivity for differentiating CIND-M from HC, compared to CIND-S; this dose-response pattern was expected given that the CIND-M classification represents deficits across multiple cognitive domains and was more likely to include impairments in executive function. Findings from the logistic regression models also speak to the known lability of cognitive impairment classifications. Specifically, the clinical trajectory of CIND is frequently recognized as unstable and heterogeneous, with several studies demonstrating that single-domain cognitive impairment classifications (e.g., CIND-S) are associated with higher instability and increased likelihood of reverting to HC compared to multi-domain classifications (e.g., Diniz et al., 2009; Loewenstein et al., 2009; Ritchie and Tuokko, 2010; Sachdev et al., 2013).

### Associations Between Short- and Long-Term Change

The ability for retest effects to be leveraged as early indicators of cognitive decline is predicated upon having accurately parameterized retest-effect-related variance, as well as the idea that short-term retest occasions are associated with long-term developmental trajectories. As some theorists have argued, short intervals may only serve as estimates of retest effects in longitudinal designs when the associations between short- and long-term change are positive and at least moderately strong. For example, Salthouse’s (2013) reported how short- and long-term changes across several cognitive domains were negatively correlated, whereby individuals showing the greatest short-term gains (between first and second sessions within one occasion) also exhibited the largest longer-term losses (across occasions). On the basis of such negative associations, it has been questioned whether short-term slopes can be reliably used as estimates of retest effects (or correspondingly as individual-differences predictors) in longitudinal models. Notably, however, Salthouse’s (2013) criticism was based upon a two-level latent change analysis (assessments within persons) of three-level nested data (sessions within occasions within persons)—a fact that raises concerns about the impact of between-context dependency on the direction, magnitude, and significance of the reported short- and long-term change estimates (estimated separately as two-level structures as opposed to derived simultaneously in three-level models) as well as their negative association.

To circumvent these concerns, in the present study we employed a novel three-level approach that more accurately parameterized short- and long-term change estimates, prior to deriving unbiased estimates of the association between retest and developmental change. For CRT RTI, we found a significant positive correlation between short- and long-retest intervals. This moderate correlation is indicative of shifts in the rank-order association between changes in short- vs. long-term CRT RTI change estimates, with the lack of significant short-term retest effects presaging the non-significant predictivity of Year 4 and 8 cognitive status. For BRT RTI, we identified a large-magnitude positive correlation between short- and long-term intervals; those who exhibited greater increases in variability across short-term retests (i.e., benefitted less from practice) also exhibited greater annualized developmental increases in RTI (a known indicator of various deleterious, age-related outcomes; MacDonald and Stawski, 2015). The increased association shown in BRT RTI further supports the potential utility of modeling short-term intervals as retest effects in longitudinal models, and is consistent with the reported susceptibility of BRT to retest-related effects (Bielak et al., 2010; Grand et al., 2016). This correlation also corroborates our logistic regression results, where individuals who benefited more from practice were also more likely to be cognitively intact at long-term follow-up. For both CRT and BRT RTI, the association between retest and developmental change slopes was positive and robust. These results are in keeping with the findings of other researchers who have advocated for the utility of short-term intervals as a proxy for retest effects and identified robust positive correlations between short- and long-term change (Zimprich et al., 2004; Hoffman et al., 2011).

### Implications for Aging Neuroscience

Our results highlight several notable implications for research on cognitive aging and the cognitive neuroscience of aging: (1) increases in RTI, even on simple psychomotor tasks, are associated with an increased risk of cognitive impairment up to four to eight years post-baseline assessment; (2) long-term developmental trajectories in cognition, while not substantially different from short-term trajectories, yield larger odds of being subsequently classified as cognitively impaired; and (3) individuals who not only fail to benefit from expected retest-related gains but also worsen in performance across years are at increased odds of being classified as cognitively impaired at follow-up. This latter result is consistent with previous literature asserting that retest effects can be a useful indicator of cognitive decline (Duff et al., 2011, 2012; Jutten et al., 2020). In the present study, the predictive utility of short- and long-term slope estimates to independently discriminate among cognitive status groups, even as many as four years later, speaks to the promise of individual differences in change for distinct time structures as predictors of future cognitive impairment. By combining modern design and analytics, researchers can systematically disaggregate short- from long-term within-person variability and utilize unbiased estimates of retest and developmental change to predict cognitive health and impairment. By using retest effects as a proxy for cognitive health, practitioners and individuals may be able to track inconsistencies across short-term temporal intervals, reducing the need for rigorous annual cognitive neuropsychological testing batteries. Harnessing the predictive validity of retest effects, by accurately parsing them from developmental effects, can serve as a clinically useful, non-invasive, and inexpensive tool for earlier detection and increasing diagnostic accuracy of cognitive impairment. Appropriate forethought and parameterization of retest effects are therefore paramount to both reduce systematic bias in longitudinal trend estimates as well as harness the unique opportunity that retest effects offer as individual-differences predictors.

### Study Strengths and Limitations

The current study showcases several strengths including the exploration of differing psychomotor tasks based on lower and higher-order cognitive demands (i.e., CRT vs. BRT), sufficient sample sizes for each cognitive status classification, the 8-year duration of the study permitting the examination of cognitive impairment status for both near and distal follow-up periods, as well as the use of performance variability (i.e., RTI) which has been suggested to serve as an important proxy of CNS integrity (Halliday et al., 2017). The present findings replicate previous research on the clinical utility of RTI (e.g., MacDonald and Stawski, 2020), as well as the predictive utility of retest effects over shorter intervals (e.g., Duff et al., 2012; Jutten et al., 2020) as early markers of shifts in cognitive health. Furthermore, previous researchers have suggested the use of multilevel modeling and intensive repeated measures burst designs for addressing retest effects in developmental research (e.g., Nesselroade, 1991; Sliwinski, 2008; Salthouse and Nesselroade, 2010; Sliwinski et al., 2010a; Salthouse’s, 2013); this study is among the first to combine such intricate design recommendations along with appropriately matching quantitative analyses (three-level multilevel modeling) for deriving unbiased estimates of retest and their corresponding prediction of cognitive status.

To be sure, this study is not without limitations. First, cognitive status was determined using a battery of neuropsychological measures and a distributional CIND classification, rather than by clinical interview. Additionally, cognitive status classifications were determined by performance (below 1.5 SDs based on age and education-matched peers) on the number of tasks; classifications were not determined by the nature of cognitive impairment (i.e., amnestic vs. non-amnestic) and therefore this study could not address etiology-specific impairment subtypes (MacDonald and Stawski, 2020). Second, the sample was fairly homogeneous and composed of relatively healthy, well-educated individuals which may restrict generalizability. Notwithstanding, we were able to distinguish between cognitive subgroups in this sample which highlights the robustness of our findings. It is likely that a more heterogeneous, less healthy sample would produce even stronger results. It is also recommended that additional research employ this design and modeling approach to prospectively identify whether individual differences in retest slopes can predict cognitive impairment or dementia progression, without* a priori* knowledge of cognitive groupings. Finally, our model’s long-term developmental change estimates may remain biased by retest, given that mere-exposure effects have been shown to exert influence even across longer retest intervals spanning years (Rabbitt et al., 2004; Salthouse et al., 2004). However, the significant predictive ability of our individual developmental slope estimates for BRT RTI in identifying individuals at risk of being CIND-S and CIND-M at long-term follow-ups highlights the utility of these slopes as predictors of cognitive status, irrespective of whether corresponding long-term increases in RTI are slightly underestimated due to generalized practice effects that span much longer retest intervals.

### Future Directions

Investigators seeking to further explore dissociable patterns between retest and development should consider modeling non-linear trends across short- and long-term trajectories. Additionally, exploring whether retest effects can significantly predict subtypes of CIND (e.g., non-amnestic vs. amnestic CIND) will further elucidate the utility of retest effects as sensitive indicators of cognitive decline. Finally, whereas the present study focused on RTI, future investigations may utilize our approach to explore the dissociable patterns of retest and developmental change using other common metrics (e.g., central tendency, accuracy) for cognitive function.

## Conclusions

The present study overviews an innovative approach for parameterizing retest effects in longitudinal designs where developmental outcomes in older adulthood are of interest. We leveraged an intensive repeated measurement burst design as well as three-level multilevel modeling to operationalize retest and developmental change directly and distinctly in the same model. Such an approach generates more definitive, less confounded trajectories of change by disaggregating within-person short- and long-term cognitive performance estimates. Further, when investigating the predictive utility of short- and long-term change in cognitive variability, we demonstrated that both retest effects and developmental change estimate each independently predicted cognitive status, thereby highlighting their potential clinical utility as well as underscoring the importance of accurately parameterizing both retest and developmental change in longitudinal designs. Specifically, for measures implicating executive functioning (i.e., BRT), individuals who fail to benefit from the expected influence of retest and instead exhibit both short- and long-term increases in RTI are at an increased risk of being classified as cognitively impaired up to 4 years post data collection. Researchers and clinicians alike may adopt the synergistic advantages of the measurement burst design and three-level multilevel modeling to facilitate better parameterization of retest and developmental effects and improved predictivity of cognitive function. In doing so, retest effects may serve as a clinically useful tool for predicting prospective cognitive status without the need for overly long or intensive neuropsychological testing batteries.

## Data Availability Statement

The data analyzed in this study is subject to the following licenses/restrictions: participants of this study did not agree for their data to be shared publicly, so supporting data is not available. Requests to access these datasets should be directed to smacd@uvic.ca.

## Ethics Statement

The studies involving human participants were reviewed and approved by University of Victoria Human Research Ethics Board. The patients/participants provided their written informed consent to participate in this study.

## Author Contributions

NT, CM, and SM all contributed to the conceptual design and implementation of the research. SM reviewed and edited the manuscript and supervised the study. All authors contributed to the article and approved the submitted version.

## Conflict of Interest

The authors declare that the research was conducted in the absence of any commercial or financial relationships that could be construed as a potential conflict of interest.

## Publisher’s Note

All claims expressed in this article are solely those of the authors and do not necessarily represent those of their affiliated organizations, or those of the publisher, the editors and the reviewers. Any product that may be evaluated in this article, or claim that may be made by its manufacturer, is not guaranteed or endorsed by the publisher.
